# Supplementary material for: Potent but transient immunosuppression of T-cells is a general feature of CD71+ erythroid cells
Source: Commun Biol. 2021 Dec 10;4:1384. doi: 10.1038/s42003-021-02914-4 (PMC8664950; doi:10.1038/s42003-021-02914-4)
Supplement: Supplementary file 3 — Description of Additional Supplementary Files [file 42003_2021_2914_MOESM3_ESM.pdf]

Description of Additional Supplementary Files

File name: Supplementary Data 1.
Description: Individual clinical data of anemic and non-anemic patients.

File name: Supplementary Data 2.
Description: Source data for main manuscript and supplementary information graphs and charts together with scans of the original uncropped gels and blots.
