# Supplementary material for: Potent but transient immunosuppression of T-cells is a general feature of CD71+ erythroid cells
Source: Commun Biol. 2021 Dec 10;4:1384. doi: 10.1038/s42003-021-02914-4 (PMC8664950; doi:10.1038/s42003-021-02914-4)
Supplement: Supplementary file 6 — Reporting Summary [file 42003_2021_2914_MOESM6_ESM.pdf]

nature research

DBRP-COMMSBIO-21-0402A
Corresponding author(s): Jakub Golab and Dominika Nowis

Last updated by author(s): Nov 3, 2021

Reporting Summary

Nature Research wishes to improve the reproducibility of the work that we publish. This form provides structure for consistency and transparency in reporting. For further information on Nature Research policies, see our Editorial Policies and the Editorial Policy Checklist.

Statistics

For all statistical analyses, confirm that the following items are present in the figure legend, table legend, main text, or Methods section.

n/a	Confirmed	
☐	☒	The exact sample size (*n*) for each experimental group/condition, given as a discrete number and unit of measurement
☐	☒	A statement on whether measurements were taken from distinct samples or whether the same sample was measured repeatedly
☐	☒	The statistical test(s) used AND whether they are one- or two-sided<br>*Only common tests should be described solely by name; describe more complex techniques in the Methods section.*
☐	☒	A description of all covariates tested
☐	☒	A description of any assumptions or corrections, such as tests of normality and adjustment for multiple comparisons
☐	☒	A full description of the statistical parameters including central tendency (e.g. means) or other basic estimates (e.g. regression coefficient) AND variation (e.g. standard deviation) or associated estimates of uncertainty (e.g. confidence intervals)
☐	☒	For null hypothesis testing, the test statistic (e.g. F, t, r) with confidence intervals, effect sizes, degrees of freedom and P value noted<br>*Give P values as exact values whenever suitable.*
☒	☐	For Bayesian analysis, information on the choice of priors and Markov chain Monte Carlo settings
☒	☐	For hierarchical and complex designs, identification of the appropriate level for tests and full reporting of outcomes
☒	☐	Estimates of effect sizes (e.g. Cohen's *d*, Pearson's *r*), indicating how they were calculated

Our web collection on statistics for biologists contains articles on many of the points above.

Software and code

Policy information about availability of computer code

Data collection

Flow cytometry data was collected with FACSDiva software (BD Biosciences).
Blots were imaged using ChemiDoc Touch Gel Imaging System (Bio-Rad).
Absorbance was measured using Asys UVW 340 Plate Reader.
Data was collected in Excel (Microsoft).

Data analysis

Graphpad Prism 8.4.3 (GraphPad Software) was used for statistical analyses. Data distribution was tested using the Shapiro-Wilk test, D'Agostino & Pearson test, and Kolmogorov-Smirnov test. Statistical analyses of three or more groups were compared using one-way analysis of variance (ANOVA) or Brown-Forsythe ANOVA followed by Tukey's, Dunnett's, or Bonferroni's multiple comparisons test or Kruskal-Wallis test followed by Dunn's multiple comparisons test. Repeated measures ANOVA with Sidak's or Holm-Sidak's post-hoc tests were used to analyze the differences in paired samples. Statistical analyses of two groups were compared using unpaired t-test, paired t-test, or Mann-Whitney test. Methods of statistical analyses are defined in every figure legend. P-value of less than 0.05 was statistically significant. Each experiment was performed in technical duplicates or triplicates. The number of biological replicates for each experiment is mentioned in the figure legends.
For flow cytometry, FlowJo v10.6.1 software (BD Biosciences) or BD FACSDiva software (BD Biosciences) was used, followed by statistical analysis performed in Graphpad Prism 8.4.3.
Molecular docking of phenylhydrazine in Arg1 and Arg2 was carried out with GOLD (Jones et al. 1997) and Surflex (Jain 2003). Simulation of phenylhydrazine binding to arginases was carried out with Gromacs (Pronk et al. 2013).
Densitometry analysis were performed using ImageJ (National Institutes of Health USA).

For manuscripts utilizing custom algorithms or software that are central to the research but not yet described in published literature, software must be made available to editors and reviewers. We strongly encourage code deposition in a community repository (e.g. GitHub). See the Nature Research guidelines for submitting code & software for further information.

Data

Policy information about availability of data

All manuscripts must include a data availability statement. This statement should provide the following information, where applicable:

- Accession codes, unique identifiers, or web links for publicly available datasets
- A list of figures that have associated raw data
- A description of any restrictions on data availability

Arginases structure were obtained from RCSB Protein Data Bank (pdb|4hww for Arg1 and pdb|4hze for Arg2)
Source data is provided for Fig.1b-d,f,h,i; Fig. 2b,d; Fig. 3a-g, i-k ; Fig.4a,b,d; Fig. 5a-n; Fig. 6a-c; Fig.7a, c-f, h; Fig.8b,c,e,f; Fig.9b-d, f-h;Fig.10b, c, f-j; Supplementary Fig. 1a-g; Supplementary Fig. 2a-b; Supplementary Fig. 3a, Supplementary Figure 3d-g; Supplementary Fig. 5b-f; Supplementary Fig. 6d-g; Supplementary Fig. 7a,b; Supplementary Fig. 8a,b; Supplementary Fig. 10a-c; Supplementary Fig. 11; Supplementary Fig. 12; Supplementary Fig. 13b,c; Supplementary Fig. 14a-c; Supplementary Fig. 15b,c,e; Supplementary Fig. 16b,e,f; Supplementary Fig. 17a; Supplementary Fig. 18a,b; Supplementary Fig. 21d,e; Supplementary Fig. 22a-c; Supplementary Fig. 23a,b; Supplementary Fig. 24b, 24d-e; Fig. 25b,c.

Field-specific reporting

Please select the one below that is the best fit for your research. If you are not sure, read the appropriate sections before making your selection.

☒ Life sciences ☐ Behavioural & social sciences ☐ Ecological, evolutionary & environmental sciences

For a reference copy of the document with all sections, see nature.com/documents/nr-reporting-summary-flat.pdf

Life sciences study design

All studies must disclose on these points even when the disclosure is negative.

Sample size	No sample-size calculations were performed. Sample size was determined to be adequate based on the degree and consistency of differences between groups
Data exclusions	No data were excluded from the analyses.
Replication	All experiments were confirmed using technical and biological replicates. Number of biological replicates is defined in the figure legends.
Randomization	Mice in the close ages and similar weight were randomly assigned to experimental and control groups.
Blinding	The investigators involved in this study were not blinded during data collection and/or analysis.

Reporting for specific materials, systems and methods

We require information from authors about some types of materials, experimental systems and methods used in many studies. Here, indicate whether each material, system or method listed is relevant to your study. If you are not sure if a list item applies to your research, read the appropriate section before selecting a response.

Materials & experimental systems

n/a	Involved in the study	
☐	☒	Antibodies
☐	☒	Eukaryotic cell lines
☒	☐	Palaeontology and archaeology
☐	☒	Animals and other organisms
☐	☒	Human research participants
☒	☐	Clinical data
☒	☐	Dual use research of concern

Methods

n/a	Involved in the study	
☒	☐	ChIP-seq
☐	☒	Flow cytometry
☒	☐	MRI-based neuroimaging

Antibodies

Antibodies used	Fluorophore- or biotin-conjugated antibodies specific for mouse cell-surface antigens and cytokines were as follows: anti-CD71 (8D3, NovusBio; R17217, eBioscience), anti-TER119 (TER-119, BioLegend), anti-CD45.2 (104, BD Biosciences), anti-CD45 (30-F11, BioLegend), anti-CD44 (IM7, BioLegend), anti-CD3e (145-2C11, eBioscience), anti-CD4 (GK1.5, eBioscience; RM4-5. eBioscience), anti-CD8a (53-6.7, eBioscience), anti-CD69 (H1.2F4, eBioscience), anti-CD25 (PC61.5, eBioscience), anti-CD62L (MEL-14, Invitrogen), anti-CD11b (M1/70, BioLegend), anti-CD11c (HL3, BD Bioscience) anti-CD3 zeta (H146-968, Abcam), anti-IFN-γ (XMG1.2, eBioscience), anti-TNF-α (MP6-XT22, eBioscience), anti-Arg1 (polyclonal, IC5868P/F, R&D Systems), anti-Arg2 (ab81505, Abcam), goat anti-rabbit IgG (Invitrogen).<br>Fluorophore- or biotin-conjugated antibodies specific for human cell-surface antigens and cytokines were as follows: anti-CD71

(CY1G4, BioLegend, DF1513, NovusBio), anti-CD235a (HI264, BioLegend), anti-CD44 (IM7, BioLegend), anti-CD45 (HI30, BD Bioscience), anti-CD49d (9F10, eBioscience), anti-CD36 (NL07, eBioscience), anti-CD34 (561, BioLegend), anti-CD25 (BC96, eBioscience), anti-CD69 (FN50, eBioscience), anti-CD3 (OKT3, eBioscience), anti-CD4 (RPA-T4, eBioscience), anti-CD8a (RPA-T8, eBioscience), anti-IFN-γ (4S.B3, BioLegend), anti-TNF-α (MAb11, BD Bioscience), anti-Arg1 (polyclonal, IC5868P/F, R&D Systems), anti-Arg2 (ab137069, Abcam), goat anti-rabbit IgG (Invitrogen).

Validation

All antibodies used in this study are commercially available. Antibodies used in a specific species or application have been appropriately validated by manufacturers for that application and this information is provided on their website and product information datasheets. Anti-Arg2 antibody was validated for flow cytometry using positive and negative controls.

Eukaryotic cell lines

Policy information about cell lines

Cell line source(s)

K562 (ATCC® CCL-243™), HEL92.1.7 (ATCC® TIB-180™) and TF-I (ATCC® CRL-2003™) cell lines were purchased from American Type Culture Collection (ATCC).

Authentication

Cells were not authenticated.

Mycoplasma contamination

Tumor cell lines were regularly tested for Mycoplasma spp. contamination and confirmed to be negative.

Commonly misidentified lines
(See ICLAC register)

No commonly misidentified cell lines were used.

Animals and other organisms

Policy information about studies involving animals; ARRIVE guidelines recommended for reporting animal research

Laboratory animals

C57BL/6 both male and female 8-week-old to 14-week-old mice were obtained from the Animal House of the Polish Academy of Sciences, Medical Research Center (Warsaw, Poland). B6.129S4-Arg1tm1Lky/J (YARG), Arg2tm1Weo/J (Arg2 functional knockout, Arg2-/-) and C57BL/6-Tg(TcraTcrb) 1100Mjb/J (OT-I) mice were purchased from the Jackson Laboratories and bred at the animal facility of the Department of Immunology, Medical University of Warsaw. Mice were housed in controlled environmental conditions in specific-pathogen-free (SPF) conditions (breeding cages, OT-I mice) or conventional (others) animal facility of the Department of Immunology, Medical University of Warsaw, with water and food provided ad libitum.

Wild animals

The study did not involve wild animals.

Field-collected samples

The study did not involve samples collected from the field.

Ethics oversight

The experiments were performed in accordance with the guidelines approved by the II Local Ethics Committee in Warsaw (approval No. WAW2/117/2019 and WAW2/143/2020) and in accordance with the requirements of EU (Directive 2010/63/EU) and Polish (Dz. U. poz. 266/15.01.2015) legislation.

Note that full information on the approval of the study protocol must also be provided in the manuscript.

Human research participants

Policy information about studies involving human research participants

Population characteristics

Peripheral blood samples were obtained from patients hospitalized in the Central Teaching Clinical Hospital, Medical University of Warsaw or treated in the Outpatient Clinic of Central Teaching Clinical Hospital, Medical University of Warsaw, Warsaw. The study was conducted in accordance with the Declaration of Helsinki. Study was approved by the Bioethical Committee of Medical University of Warsaw (KB/8/2021). Men and women aged 16-90 (mean 58.5) were included for the study. Patients with proliferative diseases, including cancer, or with hemolytic anemia were excluded from the study. Human bone marrow aspirates from healthy donors were commercially obtained from Lonza. Bone marrow donors were both males (n=6) and females (n=3) at the age of 23-45. Mobilized peripheral blood stem cells (PBSCs) were obtained from familial donors from the material remaining after allogeneic stem cell transplantation. Informed consent was obtained from the PBSC cell donors.

Recruitment

Peripheral blood samples were obtained from patients with or without anemia based on WHO diagnostic criteria. Patients with proliferative diseases, including cancer, or with hemolytic anemia were excluded from the study. Blood samples with blood hemolysis were excluded from the analysis.
Human bone marrow aspirates from healthy donors were commercially obtained from Lonza. Recrutiment was performed by Lonza. US-based donors between the ages of 23 and 45 years old. Samples were collected after obtaining permission for their use in research applications by informed consent or legal authorization. All donors were screened for general health and negative medical history for heart disease, kidney disease, liver disease, cancer, epilepsy, and blood or bleeding disorders. All donors had negative clinical laboratory tests for HIV-1, HIV2, hepatitis B, and hepatitis C.

Ethics oversight

The study was conducted in accordance with the Declaration of Helsinki. Study was approved by the Bioethical Committee of Medical University of Warsaw (KB/8/2021).

Note that full information on the approval of the study protocol must also be provided in the manuscript.

Flow Cytometry

Plots

Confirm that:

☒ The axis labels state the marker and fluorochrome used (e.g. CD4-FITC).

☒ The axis scales are clearly visible. Include numbers along axes only for bottom left plot of group (a 'group' is an analysis of identical markers).

☒ All plots are contour plots with outliers or pseudocolor plots.

☒ A numerical value for number of cells or percentage (with statistics) is provided.

Methodology

Sample preparation	Flow cytometry was performed on FACSCanto II (BD Biosciences) or Fortessa X20 (BD Biosciences) operated by FACSDiva software. Spleens were harvested and single-cell suspension was prepared. Fluorochrome-conjugated antibodies used for the staining are listed above. For cell surface staining, cells were stained with Zombie NIR™ or Zombie Aqua™ Fixable Viability Kit (Biolegend), blocked on ice with 5% normal rat serum in FACS buffer (PBS; 1% BSA, 0.01% sodium azide) and then incubated for 30 min on ice with fluorochrome-labelled antibodies. After washing in FACS buffer, cells were immediately analysed. For intracellular staining, membrane-stained cells were fixed using Fixation Buffer for 30 min, followed by wash with permeabilization buffer, and staining with antibody diluted in permeabilization buffer for 30 min (Intracellular Fixation & Permeabilization Buffer Set, eBioscience). For anti-Arg2 indirect intracellular staining, cells were fixed using Fixation Buffer for 30 min, followed by wash with permeabilization buffer, and staining with anti-Arg2 antibody for 1h, followed by wash with permeabilization buffer and staining with fluorochrome-conjugated goat anti-Rabbit IgG for 30 min.
Instrument	FACSCanto II (BD Biosciences) or Fortessa X20 (BD Biosciences)
Software	FACSDiva 6.1.3 (FACSCanto II) or FACSDiva 8 (Fortessa X20) software<br>Flow cytometry data analysis was conducted using FlowJo Version V10.6.1 software (TreeStar).
Cell population abundance	Cell frequencies are expressed as the percentage of the parent population.<br>EPCs purity after CD71+ cells positive selection was determined using flow cytometry and was >80%. Purity of CD4+ or CD8+ T-cells after negative selection was >80%.
Gating strategy	In general, the gating strategy used for all relevant experiments started with a preliminary FSC (linear)/SSC (linear) gate around the cell population, excluding very low FSC/SSC events which are expected to be dead cells, nuclei or debris. Single cells were selected by gating at FSC-A (linear) vs FSC-H (linear) excluding doublets or larger aggregates. Live cells were selected by gating as APC-Cy7 (Zombie NIR), INDO1 (Zombie UV) or V450 (Zombie Aqua) negative. Exact gating strategies for all figures are provided in the Supplementary Information.

☒ Tick this box to confirm that a figure exemplifying the gating strategy is provided in the Supplementary Information.
